# The Google Health Digital Well-Being Study: Protocol for a Digital Device Use and Well-Being Study

**DOI:** 10.2196/49189

**Published:** 2024-05-14

**Authors:** Daniel McDuff, Andrew Barakat, Ari Winbush, Allen Jiang, Felicia Cordeiro, Ryann Crowley, Lauren E Kahn, John Hernandez, Nicholas B Allen

**Affiliations:** 1 Google Mountain View, CA United States; 2 University of Oregon Eugene, OR United States

**Keywords:** digital, health, well-being, mobile, google health, digital health, well-being, mhealth, digital device, smartphone

## Abstract

**Background:**

The impact of digital device use on health and well-being is a pressing question. However, the scientific literature on this topic, to date, is marred by small and unrepresentative samples, poor measurement of core constructs, and a limited ability to address the psychological and behavioral mechanisms that may underlie the relationships between device use and well-being. Recent authoritative reviews have made urgent calls for future research projects to address these limitations. The critical role of research is to identify which patterns of use are associated with benefits versus risks and who is more vulnerable to harmful versus beneficial outcomes, so that we can pursue evidence-based product design, education, and regulation aimed at maximizing benefits and minimizing the risks of smartphones and other digital devices.

**Objective:**

The objective of this study is to provide normative data on objective patterns of smartphone use. We aim to (1) identify how patterns of smartphone use impact well-being and identify groups of individuals who show similar patterns of covariation between smartphone use and well-being measures across time; (2) examine sociodemographic and personality or mental health predictors and which patterns of smartphone use and well-being are associated with pre-post changes in mental health and functioning; (3) discover which nondevice behavior patterns mediate the association between device use and well-being; (4) identify and explore recruitment strategies to increase and improve the representation of traditionally underrepresented populations; and (5) provide a real-world baseline of observed stress, mood, insomnia, physical activity, and sleep across a representative population.

**Methods:**

This is a prospective, nonrandomized study to investigate the patterns and relationships among digital device use, sensor-based measures (including both behavioral and physiological signals), and self-reported measures of mental health and well-being. The study duration is 4 weeks per participant and includes passive sensing based on smartphone sensors, and optionally a wearable (Fitbit), for the complete study period. The smartphone device will provide activity, location, phone unlocks and app usage, and battery status information.

**Results:**

At the time of submission, the study infrastructure and app have been designed and built, the institutional review board of the University of Oregon has approved the study protocol, and data collection is underway. Data from 4182 enrolled and consented participants have been collected as of March 27, 2023. We have made many efforts to sample a study population that matches the general population, and the demographic breakdown we have been able to achieve, to date, is not a perfect match.

**Conclusions:**

The impact of digital devices on mental health and well-being raises important questions. The Digital Well-Being Study is designed to help answer questions about the association between patterns of smartphone use and well-being.

**International Registered Report Identifier (IRRID):**

DERR1-10.2196/49189

## Introduction

The impact of digital device use on health and well-being is a pressing question to which individuals, families, schools, policy makers, legislators, and digital designers are all demanding answers [1]. However, the scientific literature on this topic to date is marred by small and unrepresentative samples, poor measurement of core constructs (eg, device use and smartphone addiction), and a limited ability to address the psychological and behavioral mechanisms that may underlie the relationships between device use and well-being [2-5]. A number of authoritative reviews have made urgent calls for future research projects to address these limitations [6-9]. The critical role of the research is to identify which patterns of use are associated with benefits versus risks and who is more vulnerable to harmful versus beneficial outcomes, so that we can pursue evidence-based product design, education, and regulation aimed at maximizing benefits and minimizing the risks of smartphones and other digital devices.

Leveraging objective data from smartphones and wearables is needed to advance scientific research in this area. Physiological and behavioral biomarkers that can be measured by smartphones and wearables present a valuable way of identifying those at risk and measuring risk factors. Promising prior research has found that longitudinal smartphone and wearable data can be used to predict and, perhaps more importantly, complement existing clinical measures [7,10,11]. Indeed, the variables that can be captured by these devices can help us understand some of the most important determinants of health. For example, behavioral risk factors such as poor mental health, sleep, and physical activity are the greatest predictor of preventable illness and death [12], and nearly 8% of the US gross domestic product is lost to mental disorders each year [13]. However, mental and behavioral health conditions are complex and multifaceted phenomena, and measurement is nontrivial. Illness can manifest in a wide range of symptoms [14] that impact both physiological states and behavior. While there are examples of impressive longitudinal studies [7,15], the scientific literature on these topics is still dominated by studies with small and often unrepresentative samples, poor measurement of core constructs (eg, device use), self-reported measures of behavior that may not correlate with objectively measured end points, and a limited ability to address the psychological and behavioral mechanisms that may underlie the relationships between device use and well-being. For example, some studies focus solely on college students [15] or medical interns [7]. A number of recent authoritative reviews have made urgent calls for future research projects to address these limitations [7-9,16].

To address this gap in the literature, we have designed a large-scale intensive longitudinal study (the Digital Well-Being [DWB] study) to analyze the relationships among smartphone use, physiological and behavioral data from wearables and smartphones, and self-reported measures of mental health and well-being. We will recruit up to 14,000 participants who opt-in to complete intake and outtake surveys and daily ecological assessments and enable sensing of features from their smartphone. In addition, at least 50% (n=7000) of the participants will wear a smartwatch (Fitbit). Each individual’s participation in the study will be for 28 days.

The aims of the DWB study are manifold. We aim to (1) provide normative data on objective patterns of smartphone use and describe how these patterns of use vary with age, gender, geography, and functioning or mental health; (2) identify how patterns of smartphone use impact well-being and who is most vulnerable to harmful effects related to smartphone use; (3) identify groups of individuals who show similar patterns of covariation between smartphone use and well-being measures across time; (4) examine sociodemographic and personality or mental health predictors; (5) examine which patterns of smartphone use and well-being are associated with pre-post changes in mental health and functioning and compare the predictive capacity of these objective measures to relevant self-report measures (eg, smartphone addiction measures); (6) discover which nondevice behavior patterns (eg, sleep, physical activity, geographic movement, and social interactions) mediate the association between device use and well-being; (7) identify and explore recruitment strategies to increase and improve the representation of traditionally underrepresented populations; and (8) provide a real-world baseline of observed stress, mood, insomnia, physical activity, and sleep across a representative population.

Next, the methods used in our data collection protocol and preliminary statistics on the demographic profile of the first cohort of participants will be described.

## Methods

### Study Design

**Figure 1:**
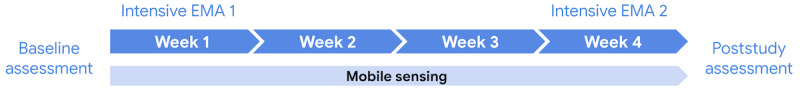
The design of the 4-week intensive digital sensing study. Participants will complete baseline and poststudy assessments, and the first and last weeks of the study involves daily intensive ecological momentary assessments. Mobile sensing consisting of measurements from a smartphone, and optionally a wrist-worn wearable, will be conducted throughout the study. EMA: ecological momentary assessment.

The DWB study is a prospective, nonrandomized study to investigate the patterns and relationships between digital device use patterns, including sensor data from phones and wearables reflecting both behavioral and physiological processes and self-reported measures of mental health and well-being. The study is 4 weeks long with passive sensing from a smartphone, and optionally a wearable (Fitbit), for the complete study period (Figure 1).

### Recruitment

#### Overview

A key goal of the study is to explore methods to collect a representative study sample that reflects the relative proportions of various demographic subgroups and their intersectionalities in adults of the US population. For example, according to the US Census Bureau [17], 51% of the population is female and 44% of adults are between 20 and 44 years of age. Among those who reported a single race, about 13% are African American, 6% are Asian, and 74% are White. A February 2021 Gallup poll [18] reported that 5.6% of US adults identified themselves as lesbian, gay, bisexual, or transgender; 86.7% said that they were heterosexual or straight; and 7.6% refused to answer. In the past, some subgroups have been traditionally underrepresented in health studies (eg, female individuals; African American individuals; and those with a lesbian, gay, bisexual, transgender, queer, intersex, and asexual orientation) [19], and recent reviews have emphasized the importance of setting explicit recruitment goals in overcoming these biases [19,20]. As such, a key aim of the study is to learn more about how to increase the participation of traditionally underrepresented groups in digital health studies.

To explore methods to overcome recruitment barriers of traditionally underrepresented subgroups and make sure the study sample reflects sufficient diversity, we will pursue a two-prong approach: (1) a primary-augmented 2-wave metered recruitment schema and (2) multistakeholder engagement. Metered recruitment schema refers to the potential delay of eligible study participants in consent and enrollment (baseline characterization and initiation into the monitoring portion of the study). The pace of enrollment will be adapted based on study and clinical bandwidths and the distributions of demographic characteristics, with metering instituted by invitation to consent. The research team plans to begin the metered recruitment strategy once the study has achieved or has nearly achieved the enrollment of 10,000 participants. Depending on the distribution of key demographic variables, once consent and appropriate permissions are obtained, enrollment and initiation of monitoring will commence. To help reduce the effect of seasonality and other external factors on the data collected in this study, we are careful to design the strategy to recruit participants in a balanced manner across time and outside of periods containing major seasonal holidays.

#### Wave 1 (Open Enrollment With Enriched Recruitment)

During the initial several weeks that the study is open, we anticipate enrollment of up to approximately 1000 new participants per week (but will not limit to this pace). Enrollment will increase as infrastructure allows. An increase in consecutive enrollment of up to 10,000 new participants who are eligible for study participation will be invited to begin the enrollment process. The study will monitor the progress in recruiting traditionally underrepresented groups. Recruitment efforts will focus on enriched representations, with recruitment via email, social media, and Fitbit in-app notifications. Posts will be made on social media platforms, leveraging the reach and audience of the University of Oregon and Google accounts. In-app notifications will be made in the Fitbit app, and these notifications ensure high visibility of the study. At the conclusion of wave 1, the study investigators will assess study participation rates and revise the enrollment targets for wave 2.

#### Wave 2 (Augmented Enrollment)

Upon the completion of wave 1, wave-2 efforts will discontinue the enrollment of sufficiently represented groups and focus entirely on increasing the participation of underrepresented groups, as defined by race and ethnicity (eg, African American, Asian, Latina or Latino, Native Americans or Indigenous, and White populations), biological sex at birth (female and male), age (18-40 and over 40 years), sexual orientation or gender identity (heterosexual and lesbian, gay, bisexual, transgender, queer, intersex, and asexual), and whether participants are Fitbit users. Multistakeholder engagement refers to the study’s collaboration with major stakeholders such as active groups or key opinion leaders of underrepresented groups of interest to codevelop adaptive recruitment goals and strategies.

The study will aim to recruit a study sample that reflects enriched participation of traditionally underrepresented groups in the United States. The wave-2 targeted recruitment strategy will include 5 demographic factors in addition to the ownership of Fitbit devices. If wave-1 enrollment is not sufficiently at parity with population representation, wave-2 efforts will target up to an additional 4000 participants to demographically balance the aggregate sample (potential total n≤14,000).

### Ethical Considerations

If they choose to enroll, participants will be directed to a consent form administered via an in-app onboarding flow that describes the data that are collected and how they are used to advance the goals of the study. Each prospective study participant must pass the eligibility survey before proceeding to an assurance of understanding flow, where they are given a summary of the summary and key points of the consent. They are then quizzed on this content before they can proceed. There are multiple points to ensure comprehension of the study and consent. Prospective study participants agree to an e-consent, which provides short explanations for the data they are sharing and how they are used and a long form consent, which is a full institutional review board–approved consent form detailing the data use and rights. In addition, at the top of the form is a textbox including basic details of the study designed to make sure participants receive the most critical details, regardless of how.

The consent form will be associated with a unique participant ID that is stored securely. The purpose of this ID is to be able to link the consent form to the data collected in order to confirm that data were collected with consent if needed. As mobile sensing is nonintrusive, participants may easily forget they are participating in the study; therefore, the participants will receive daily notifications reminding them that they are enrolled in the study.

Participants will be informed that if they choose to withdraw, and they will have the option to remove or retain all or part of their data. In addition, even if they do not withdraw from the study, they may choose at any time to have any portion of their data omitted, giving the dates and times between which they want their data to be removed.

Each consent form will be read and signed electronically, since this study is entirely on the web. Participants will indicate their consent to participate in this study by inputting their name and email to indicate that they have read and understood the informed consent document. Participants will be given the option to contact the research staff if they have any concerns regarding the consent. Participants will be emailed a copy of the consent form to their email address.

Finally, the study design includes an assurance of understanding quiz made up of 8 short questions about the study procedures. This includes questions on risks, benefits, data collection, data access and security, and privacy measures. Participants could only enroll in the study if they answered all 8 questions correctly.

Passive data collection begins only after participants have completed all onboarding surveys. To initiate passive data collection, participants must complete a comprehensive permission granting process in which they grant access to each data type in turn (eg, GPS signal and activity signal). The data will be collected in a manner that inherently restricts the granularity and detail of participant information. For example, data on app usage will be bundled by hourly bucket and by category of app rather than collecting individual app names and timestamps. As another example, location information is collected semantically (eg, “home,” “work,” or “other location”) rather than precisely (eg, GPS coordinates). That is, investigators do not know where participants geographically are; instead, they only possess information regarding whether participants spent time at certain semantic locations of interest.

Data will be encrypted from the point of collection to the point of delivery in the secure database and transmitted in this fashion. Even if these data were intercepted by a nefarious actor, they would be protected by ‎Secure Hash Algorithms 256 encryption and would only be accessible to systems that have the private decryption key (only the research team).

Data will be deidentified. Although this study is outside the bounds of HIPAA (Health Insurance Portability and Accountability Act; neither party is a covered entity or a business associate of a covered entity), the research team has removed all of the 18 HIPAA identifiers (name, email, address, etc) from the study data. The data will be stored in a secure, access-restricted, and password-protected database that is audited extensively at monthly, quarterly, semiannual, and annual intervals by security, privacy, and compliance teams and processes. Access to this database is restricted strictly to members of the research team who all completed Collaborative Institutional Training Initiative training.

There are two main types of data collected in the study: (1) study data, which include all active and passive investigational data (surveys, smartphone data, and wearable data) and (2) consent responses, which contain the participant consent forms. These 2 data sets are kept entirely separate. Consent forms must be retained for liability and legal purposes. Access to these forms is restricted to members of the research team who do not have access to the study data.

The institutional review board of the University of Oregon has approved the study protocol (STUDY00000162). In addition, this study went through several rounds of ethical reviews within Google, including sign-off from the responsible artificial intelligence team; the health ethics team; and the privacy, security, and legal teams. Further, an external advisory panel was assembled with mental health experts from outside of Google to solicit their input and feedback on the study design.

We leverage a raffle design for incentives and compensation. Participants who complete the required elements of the study will be eligible to enter the raffle to receive a US $50 gift card. Specifically, the conditions for eligibility were to (1) consent and enable sensor collection at study start, (2) complete the prestudy assessments, (3) complete a minimum cumulative of 7 days (1 week) of daily status assessments, and (4) complete the poststudy assessments. We will segment the raffles in batches of 1000 participants to avoid delaying delivery of the incentive until the completion of the study (which could be quite some time after the first several thousand participants complete the participant duration of 4 weeks). Up to 1610 participants may receive this incentive over the course of the study, with an 11.5% win ratio.

### Data Collection

#### Digital Measures

**Figure 2:**
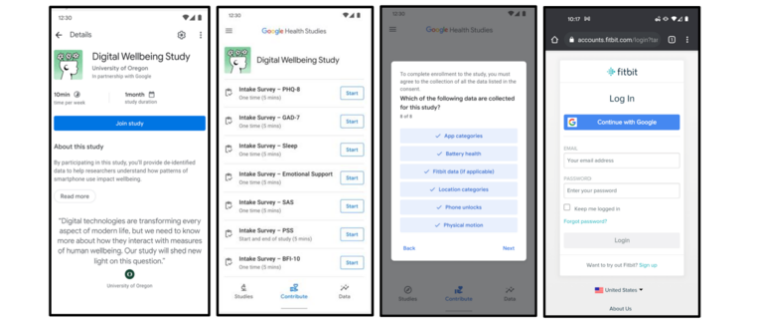
Screenshots of the Android phone app that will be used in the Digital Well-Being study. The app facilitates enrollment and consent, baseline and poststudy surveys, EMAs, and logging of the smartphone measures. Participants can optionally link their Fitbit account. EMA: ecological momentary assessment.

The study includes digital measurement from both smartphones and wearable physiological devices. After enrolling in the study, participants will be asked to grant permissions to their devices. Figure 2 shows screenshots of the phone app to illustrate the design.

#### Mobile Data

Patterns of device use will be measured objectively via the study mobile app. The measurements will include:

- Screen on time and app usage: the amount of screen time derived from smartphone app usage data such as time of day and day of week computed per app type—app usage will be quantified for categories of apps rather than individual apps and app categories will be determined by those listed on the Google Play Store.
- Mobility and semantic location information: various measures of geographic mobility will be discerned from GPS data such as daily count of travel or stop events per person per day; duration of each travel or stop event (minutes); and proportion of day spent at home, work, or some other location (labeled “other”).
- Human activity recognition from smartphone accelerometer: various movement patterns of human activities of interest such as stationary, walking, running, biking, and driving will be derived from data generated by smartphone accelerometers without being limited by phone location or orientation.
- Battery and charging status: smartphone battery status and plug-in or unplug events.

#### Wearable Data

We will collect the following data from participants who sign up with Fitbit devices. As access to devices can vary, participation is allowed with any generation of Fitbit devices. Different Fitbit devices have different sensors and algorithmic capabilities, and therefore not all the measures described below will be available for all participants. Minute-level features include accelerometer-based measures of steps and photoplethysmography-based heart rate measurements. Day-level features include accelerometer-based measures of activity (eg, total steps, number of floors climbed, and number of active zone minutes), sleep stages, duration and quality, minutely heart rate, daily resting heart rate, respiration rate during sleep, heart rate variability during sleep, skin temperature during sleep, blood oxygen saturation during sleep, and tonic electrodermal activity.

#### Survey Instruments

Participants will complete a battery of self-report questionnaires at the beginning (baseline) and end of the study (poststudy) as follows: (baseline only) demographics questionnaire and Big Five Inventory [21,22], Patient Health Questionnaire [21,23], Generalized Anxiety Disorder Scale [24], Patient-Reported Outcomes Measurement Information System Sleep Disturbance and Sleep-Related Impairment short form [25,26], Patient-Reported Outcomes Measurement Information System Emotional Support Short Form 4a [27,28], Shortened Smartphone Addiction Scale [29], and Perceived Stress Scale [27].

#### Ecological Momentary Assessments

The first and last week of the study will include intensive ecological momentary assessments (EMAs). During these weeks, 3 times per day, participants will be sent 6 identical survey questions per day for 7 days, on a quasirandomized schedule. The first 5 questions will be “How X do you feel right now?” where X will be replaced with a specific affect descriptor from happy, calm, anxious, sad, stressed, and participants will be asked to rate each item. The final question is “Over the last few hours, who have you spent the most time with?” and participants will choose from alone, friends, family, spouse or partner, coworkers, and costudents. Finally, every day for the entire duration of the study, participants will be asked “In general, how have you been feeling over the last day?” once each morning.

#### Analysis Plan

The first phase of the hypothesis-driven data analysis will be to process the raw data signals to derive interpretable features to test explicit hypotheses. The study will test a range of specific hypotheses and it would be beyond the scope of this paper to list them all. However, we present an exemplar here to demonstrate how such hypotheses could be tested. For example, 1 hypothesis that has been proposed in the research literature is that a key mechanism by which device use may affect mental health and well-being is by disrupting healthy sleep behaviors [30]. Specifically, it has been found that device use during the prebedtime period may delay sleep onset either by displacing sleep by device use or by increasing psychological arousal prior to lights out, thereby increasing sleep onset latency. In this study, estimates of device use (in the prebedtime period) will be combined with sleep features, including bedtime and rise time, time in bed, and total sleep time. Specifically, we will test whether the hypothesized mechanisms of change (ie, sleep) mediate the relationship between device use patterns and measures of the change (slope) in measures of mental health and well-being across the study period. Mediating effects will be tested using the strategies outlined by Hayes and Preacher [31], which are standard in behavioral research trials. Using linear structural equation models, support for mediation will be determined if measures of changes in measures of mental health and well-being become nonsignificant or are significantly reduced after controlling for the hypothesized mediating variable. We will assess the joint significance of the indirect pathways (ie, the joint significance of the pathways from the predictor to the mediating variable and from the mediating variable to psychiatric outcomes) using the bias-corrected bootstrap test and will also use the bootstrapping technique to test multiple simultaneous mediators [31].

The intensive longitudinal data on these features and self-reports of well-being will be subjected to data reduction techniques such as dynamic factor analysis, which are a set of techniques for estimating common trends in multivariate time series [32]. Dynamic factor analysis is a dimension reduction technique that aims to model a multivariate observed time series in terms of a finite number of common trends, with the aim of detecting the smallest number of trends that can summarize the data without losing information. The principle is the same as in other dimension reduction techniques, such as principal component analysis and factor analysis, except that the axes are restricted to be latent smoothing functions over time. The dynamic factor analysis will initially use data from the entire 4-week data collection period to examine the patterns of association between daily patterns of device use and daily variations in well-being and then will go on to examine the data from the two 1-week burst of intensive EMA to examine within-day covariation between patterns of device use and well-being. Variables that do not load on to 1 of the identified factors, but that are of strong interest as putative risk factors based on theory or previous empirical research, will be investigated in the statistical analyses as univariate predictors.

The second phase of the statistical analyses will consist of a number of analyses to be conducted to address the aims outlined above. A range of analytic techniques will be used, including ANOVA, specialized multilevel regression techniques that have been used in previous studies of intensive longitudinal data [33], and mixed Markov models [34]. These models are appropriate for testing the hypotheses with our aims, whereby changes in well-being across time can be considered an observed state switch and analyzed using observed Markov models (also referred to as manifest or simple Markov models or Markov chains [34-36]). These methods can also be used to investigate the temporal relationship between the intensive longitudinal data streams and the optimal time lag between these measurements and state switches (ie, the time lag between a predictor and the onset of a change in well-being status), which can be investigated by using higher order models, where the current state is modeled as depending on multiple preceding states [37].

We also plan to investigate the use of neural models to create machine-learned representations in our analysis. The intensive longitudinal nature of the data collection will enable large-parameter models to be trained using unsupervised and supervised machine learning techniques. Learning with self-supervision has recently attracted increasing interest as simple pretext tasks can be used to create versatile compressed representations of data. The main advantage of these embeddings is that they provide an initialization for downstream tasks. This is a more label-efficient mechanism to train supervised models. The benefits of unsupervised or self-supervised learning techniques studying human behavior are still relatively underexplored. However, the high dimensionality and the scale of the data described in this protocol lend themselves to this type of analysis. We plan to empirically test whether leveraging these methods has a positive impact on downstream predictive performance.

Given that participants are not required to complete all surveys, missing data will be handled via established imputation or multiple imputation techniques; however, we also plan to test machine learning–based imputation using autoencoders (eg, masked autoencoders). We expect that some partial data may be unusable, with the wearable devices adherence in intensive longitudinal studies is a known issue.

## Results

At the time of submission, the study infrastructure and app have been designed and built, the institutional review board of the University of Oregon has approved the study protocol, and data collection is underway. Data from 4182 enrolled participants have been collected as of March 27, 2023.

Table 1 shows the distribution of participants enrolled in the DWB study at the time of writing. We have been able to achieve a demographic split relatively similar to the 2021 US Census data [17] in terms of race, biological sex, age, and sexual orientation.

**Figure 3:**
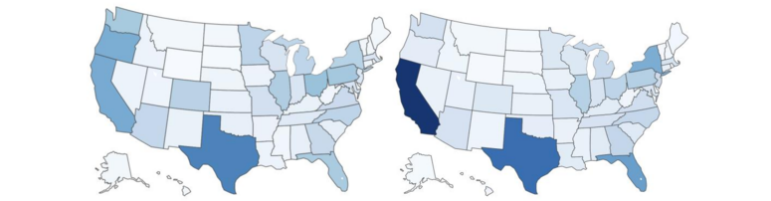
The distribution of participants by home state (in percentage). (Left) Digital Well-Being study home state density and (Right) US 2021 Census population density.

Figure 3 shows the geographic distribution of the participants based on home state. This map illustrated the geographic representation enabled by performing a study with our mobile platform and ubiquitous wearable and smartphone sensing.

**Table 1:** Comparison between our study sample and the demographic breakdown of the US adult population. Statistics as of March 27, 2023.

Factor (level)		2021 US census [17], %		Digital Well-Being study, %
**Ethnicity**				
	Hispanic or Latino	20	8	
	Not Hispanic or Latino	80	92	
**Race**				
	Asian	7	3	
	Black	14	6	
	Native American or Alaskan Native	1	1	
	White	76	82	
	Some other race	5	8	
**Biological sex**				
	Male	48	32	
	Female	48	65	
	I prefer something else	N/A^a^	3	
**Age (years)**				
	18-40	55	54	
	40+	45	46	
**Orientation**				
	Heterosexual	88	78	
	LGBTQIA+^b^	8	20	
	I prefer something else	2	2	
	I do not know	2	N/A	

^a^N/A: not available.

^b^LGBTQIA: lesbian, gay, bisexual, transgender, queer, intersex, and asexual.

## Discussion

### Principal Findings

There is a pressing need for more research into the role that digital device use plays in mental health and well-being and how these devices may be used to better monitor symptoms and complement existing clinical measures. The DWB study is an intensive longitudinal study designed to collect representative data across a large and representative population in the United States to help answer questions about the interaction between digital device use and mental health. At the time of writing, data collection is underway, with over 4000 participants enrolled.

A study of the general population means we cannot draw the same conclusions as a study of a more specific, narrow group. Unlike studies of medical interns or undergraduate students [15,38,39], where there may be some common stressors (eg, start of an internship or an examination period) that might impact all participants within a similar time frame, this study represents a much more diverse population.

Although we have made many efforts to sample a study population that matches the general population, the demographic breakdown we are able to achieve will likely not be a perfect match. This is evidenced by our study sample to date and is in part due to the desire to recruit participants with Fitbit wearable devices, a customer population that does not match the general population. It is not possible to provide participants with these devices, we therefore rely on people enrolling who have access to a Fitbit; as a result, it may be difficult to reach enough people from every demographic. Nevertheless, our initial study sample shows a promising picture that we will come close to achieving the goals.

Given the scale and nature of the study, we had to balance privacy and adherence considerations with the desire to collect highly descriptive digital data. The choice of digital probes is a product of this process. We wanted to understand sleep, mobility, physical activity, and social connectedness and, therefore, selected the digital signals informed by the desire to understand these behavioral processes. We recognize that more detailed logging (eg, app-level names) would be helpful, but these would also contain significantly more privacy sensitive data.

The Google Health DWB study will provide a valuable source of in situ behavioral and physiological observations paired with self-reported data for studying. The objective of this study is to provide normative data on objective patterns of smartphone use. To identify how patterns of smartphone use impact well-being, groups of individuals who show similar patterns of covariation between smartphone use and well-being measures across time were identified. We will examine sociodemographic and personality or mental health predictors and which patterns of smartphone use and well-being are associated with pre-post changes in mental health and functioning. These data should allow us to discover which nondevice behavior patterns (eg, sleep, physical activity, geographic movement, and social interactions) mediate the association between device use and well-being and identify and explore recruitment strategies to increase and improve the representation of traditionally underrepresented populations.

### Conclusions

A large in situ study could provide valuable insight into the role of digital device use on mental health and well-being. Through the DWB study, we intend to answer critical questions based on a large population analysis. We plan to combine measurements from wearable devices, smartphones, surveys, and EMAs. By leveraging ubiquitously available devices, we aim to sample a more representative snapshot of the US population than has previously been reported.

## Abbreviations

DWB: Digital Well-Being
EMA: ecological momentary assessment
HIPAA: Health Insurance Portability and Accountability Act

## Data Availability

The data sets generated and analyzed during this study are not publicly available.

## References

[1] Haidt J, Allen N (2020). Scrutinizing the effects of digital technology on mental health. Nature.

[2] Harris B, Regan T, Schueler J, Fields SA (2020). Problematic mobile phone and smartphone use scales: a systematic review. Front Psychol.

[3] Parry DA, Davidson BI, Sewall CJR, Fisher JT, Mieczkowski H, Quintana DS (2021). A systematic review and meta-analysis of discrepancies between logged and self-reported digital media use. Nat Hum Behav.

[4] Gerpott TJ, Thomas S (2014). Empirical research on mobile internet usage: a meta-analysis of the literature. Telecomm Policy.

[5] Griffioen N, van Rooij M, Lichtwarck-Aschoff A, Granic I (2020). Toward improved methods in social media research. Technol Mind Behav.

[6] Girela-Serrano BM, Spiers ADV, Ruotong L, Gangadia S, Toledano MB, Di Simplicio M (2022). Impact of mobile phones and wireless devices use on children and adolescents' mental health: a systematic review. Eur Child Adolesc Psychiatry.

[7] Fang Y, Forger DB, Frank E, Sen S, Goldstein C (2021). Day-to-day variability in sleep parameters and depression risk: a prospective cohort study of training physicians. NPJ Digit Med.

[8] Odgers CL, Jensen MR (2020). Annual research review: adolescent mental health in the digital age: facts, fears, and future directions. J Child Psychol Psychiatry.

[9] Orben A (2020). Teenagers, screens and social media: a narrative review of reviews and key studies. Soc Psychiatry Psychiatr Epidemiol.

[10] Zhang Y, Folarin AA, Sun S, Cummins N, Bendayan R, Ranjan Y, Rashid Z, Conde P, Stewart C, Laiou P, Matcham F, White KM, Lamers F, Siddi S, Simblett S, Myin-Germeys I, Rintala A, Wykes T, Haro JM, Penninx BW, Narayan VA, Hotopf M, Dobson RJ (2021). Relationship between major depression symptom severity and sleep collected using a wristband wearable device: multicenter longitudinal observational study. JMIR Mhealth Uhealth.

[11] Wang R, Wang W, daSilva A, Huckins JF, Kelley WM, Heatherton TF, Campbell AT (2018). Tracking depression dynamics in college students using mobile phone and wearable sensing. Proc ACM Interact Mob Wearable Ubiquitous Technol.

[12] GBD 2016 Risk Factors Collaborators (2017). Global, regional, and national comparative risk assessment of 84 behavioural, environmental and occupational, and metabolic risks or clusters of risks, 1990-2016: a systematic analysis for the Global Burden of Disease Study 2016. Lancet.

[13] Arias D, Saxena S, Verguet S (2022). Quantifying the global burden of mental disorders and their economic value. EClinicalMedicine.

[14] Galatzer-Levy IR, Bryant RA (2013). 636,120 Ways to have posttraumatic stress disorder. Perspect Psychol Sci.

[15] Xu X, Liu X, Zhang H, Wang W, Nepal S, Sefidgar Y, Seo W, Kuehn KS, Huckins JF, Morris ME, Nurius PS, Riskin EA, Patel S, Althoff T, Campbell A, Dey AK, Mankoff J (2023). GLOBEM: cross-dataset generalization of longitudinal human behavior modeling. Proc ACM Interact Mob Wearable Ubiquitous Technol.

[16] Prinstein MJ, Nesi J, Telzer EH (2020). Commentary: an updated agenda for the study of digital media use and adolescent development—future directions following Odgers and Jensen (2020). J Child Psychol Psychiatry.

[17] 2021 Releases. United States Census Bureau.

[18] Jones JM (2021). LGBT identification rises to 5.6% in latest U.S. estimate. Gallop.

[19] Oh SS, Galanter J, Thakur N, Pino-Yanes M, Barcelo NE, White MJ, de Bruin DM, Greenblatt RM, Bibbins-Domingo K, Wu AHB, Borrell LN, Gunter C, Powe NR, Burchard EG (2015). Diversity in clinical and biomedical research: a promise yet to be fulfilled. PLoS Med.

[20] Cullen MR, Lemeshow AR, Amaro S, Bandera EV, Cooper LA, Kawachi I, Lunyera J, McKinley L, Poss CS, Rottas MM, Schachterle SE, Thadeio PF, Russo LJ (2023). A framework for setting enrollment goals to ensure participant diversity in sponsored clinical trials in the United States. Contemp Clin Trials.

[21] Caprara GV, Barbaranelli C, Borgogni L, Perugini M (1993). The “big five questionnaire”: a new questionnaire to assess the five factor model. Pers Individ Differ.

[22] Rammstedt B, John OP (2007). Measuring personality in one minute or less: a 10-item short version of the big five inventory in English and German. J Res Pers.

[23] Kroenke K, Strine TW, Spitzer RL, Williams JBW, Berry JT, Mokdad AH (2009). The PHQ-8 as a measure of current depression in the general population. J Affect Disord.

[24] Löwe B, Decker O, Müller S, Brähler E, Schellberg D, Herzog W, Herzberg PY (2008). Validation and standardization of the Generalized Anxiety Disorder Screener (GAD-7) in the general population. Med Care.

[25] Cella D, Riley W, Stone A, Rothrock N, Reeve B, Yount S, Amtmann D, Bode R, Buysse D, Choi S, Cook K, Devellis R, DeWalt D, Fries JF, Gershon R, Hahn EA, Lai JS, Pilkonis P, Revicki D, Rose M, Weinfurt K, Hays R (2010). The Patient-Reported Outcomes Measurement Information System (PROMIS) developed and tested its first wave of adult self-reported health outcome item banks: 2005-2008. J Clin Epidemiol.

[26] Yu L, Buysse DJ, Germain A, Moul DE, Stover A, Dodds NE, Johnston KL, Pilkonis PA (2011). Development of short forms from the PROMIS™ sleep disturbance and sleep-related impairment item banks. Behav Sleep Med.

[27] Cohen S, Kamarck T, Mermelstein R (1983). A global measure of perceived stress. J Health Soc Behav.

[28] Bode RK, Hahn EA, DeVellis R, Cella D, Patient-Reported Outcomes Measurement Information System Social Domain Working Group (2010). Measuring participation: the patient-reported outcomes measurement information system experience. Arch Phys Med Rehabil.

[29] Kwon M, Kim DJ, Cho H, Yang S (2013). The smartphone addiction scale: development and validation of a short version for adolescents. PLoS One.

[30] Alonzo R, Hussain J, Stranges S, Anderson KK (2021). Interplay between social media use, sleep quality, and mental health in youth: a systematic review. Sleep Med Rev.

[31] Hayes AF, Preacher KJ (2010). Quantifying and testing indirect effects in simple mediation models when the constituent paths are nonlinear. Multivariate Behav Res.

[32] Zuur AF, Fryer RJ, Jolliffe IT, Dekker R, Beukema JJ (2003). Estimating common trends in multivariate time series using dynamic factor analysis. Environmetrics.

[33] Kuppens P, Allen NB, Sheeber LB (2010). Emotional inertia and psychological maladjustment. Psychol Sci.

[34] de Haan-Rietdijk S, Kuppens P, Bergeman CS, Sheeber LB, Allen NB, Hamaker EL (2017). On the use of mixed Markov models for intensive longitudinal data. Multivariate Behav Res.

[35] Kapland D (2008). An overview of Markov chain methods for the study of stage-sequential developmental processes. Dev Psychol.

[36] Langeheine R, van de Pol F, McCutcheon AL, Hagenaars JA (2002). Latent Markov chains. Applied Latent Class Analysis.

[37] Zucchini W, MacDonald IL, Langrock R (2016). Hidden Markov Models for Time Series: An Introduction Using R, Second Edition.

[38] Kalmbach DA, Fang Y, Arnedt JT, Cochran AL, Deldin PJ, Kaplin AI, Sen S (2018). Effects of sleep, physical activity, and shift work on daily mood: a prospective mobile monitoring study of medical interns. J Gen Intern Med.

[39] Taylor S, Jaques N, Nosakhare E, Sano A, Picard R (2020). Personalized multitask learning for predicting tomorrow's mood, stress, and health. IEEE Trans Affect Comput.

